# Impact of duration of structured observations on measurement of handwashing behavior at critical times

**DOI:** 10.1186/1471-2458-13-705

**Published:** 2013-08-02

**Authors:** Amal K Halder, John W Molyneaux, Stephen P Luby, Pavani K Ram

**Affiliations:** 1 International Centre for Diarrheal Disease Research, Bangladesh (icddr,b), Dhaka, Bangladesh; 2 Millennium Challenge Corporation, 875 Fifteenth Street NW, Washington DC, USA; 3 Stanford University, 473 Via Ortega, Stanford, CA, USA; 4 University at Buffalo, 3435 Main Street, Buffalo, NY, USA

## Abstract

**Background:**

Structured observation is frequently used to measure handwashing at critical events, such as after fecal contact and before eating, but it is time-consuming. We aimed to assess the impact of reducing the duration of structured observation on the number and type of critical events observed.

**Methods:**

The study recruited 100 randomly selected households, 50 for short 90-minute observations and 50 for long 5-hour observations, in six rural Bangladeshi villages. Based on the first 90 minutes in the long observation households, we estimated the number of critical events for handwashing expected, and compared the expected number to the number of events actually observed in the short observation households. In long observation households, we compared soap use at critical events observed during the first 90 minutes to soap use at events observed during the latter 210 minutes of the 5-hour duration.

**Results:**

In short 90-minute observation households, the mean number of events observed was lower than the number of events expected: before eating (observed 0.25, expected 0.45, p < 0.05) and after defecation (observed 0.0, expected 0.03, p = 0.06). However, the mean number observed was higher than the expected for food preparation, food serving, and child feeding events. In long 5-hour observation households, soap was used more frequently at critical events observed in the first 90 minutes than in the remaining 210 minutes, but this difference was not significant (p = 0.29).

**Conclusions:**

Decreasing the duration of handwashing significantly reduced the observation of critical events of interest to evaluators of handwashing programs. Researchers seeking to measure observed handwashing behavior should continue with prolonged duration of structured observation. Future research should develop and evaluate novel models to reduce reactivity to observation and improve the measurement of handwashing behavior.

## Background

Handwashing with soap can prevent infections that kill children in low- and middle-income countries [1,2]. Evaluating the behavioral impact of handwashing promotion programs is difficult because each method of measuring handwashing behavior has limitations [3].Structured observations yield detailed data on the nature and context of handwashing behavior, including whether hands are washed at specific critical events, such as after defecation [4]. Ranging from three to seven hours or more, structured observations are labor-intensive, time-consuming and expensive [4-8]. Moreover, staying at a household for a prolonged period of time may be inconvenient and uncomfortable for study participants. If the duration of structured observations could be reduced without excessive loss of data, structured observations could become more efficient since more households could potentially be observed during a work day; moreover, participation in a short duration observation might inconvenience the study participants less compared to a long observation session. To determine whether structured observations of short duration would yield data similar to observations of long duration, we compared the type and frequency of events (opportunities for handwashing) observed during short structured observations (90 minutes) to those observed during long structured observations (5 hours).To determine whether handwashing behavior is consistent over the course of a long observation period, we assessed whether handwashing behavior observed during the first 90 minutes of a 5-hour observation was consistent with behavior observed during the remaining 270 minutes. Data collection for this investigation was conducted as part of a larger study on handwashing behavior measures in rural Bangladesh [9].

## Methods

We conducted this study in six rural villages located in two districts in Bangladesh, where households are typically grouped together into clusters [9]. In each village, we enumerated 16 or 17 clusters that had at least one child less than two years old, with 5-hour observations conducted in even-numbered clusters and 90-minute observations conducted in odd-numbered clusters. The principal caregiver, typically identified as the mother, of a child less than two years old in each cluster was requested to participate in the study. In this study we used observed handwashing data of principal caregiver at households. In the long observation group, we planned for 5-hour structured observations to start between 9:00 am and 11:00 am. We sought to have field workers complete two 90-minute observations per day as a test of the efficiency of carrying out short observations. The planned start time was 9:00 am for the first set of short observations, and 12:00 pm for the second set of observations. Data collection was performed by trained female observers in July and August 2007. The observers had at least one hour break before starting the second observation of the day. Observers recorded handwashing behaviors at the following critical events using a structured checklist: preparing and serving food, feeding a child and eating, defecation, and cleaning a child who defecated. The focus of the structured observation was the primary caregiver of the child.

We obtained voluntary written informed consent from the study participants in each household. The consent document was read aloud to the study participants. The study protocol was approved by the research and ethical review committees of icddr,b and the University at Buffalo.

### Data analysis

**Figure 1:**
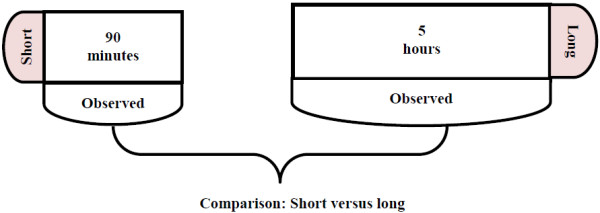
Comparisons of short and long observation groups with respect to observation of critical times, and handwashing behavior at critical times, Bangladesh, 2007.

**Figure 2:**
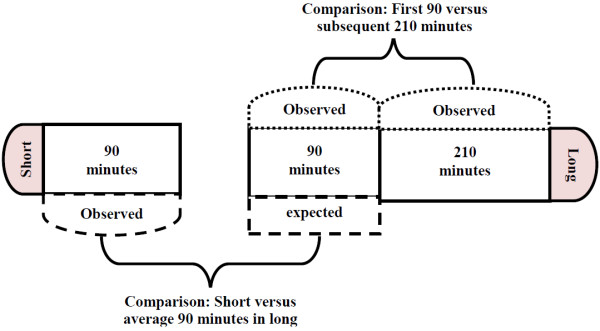
Comparisons of short and long observation groups with respect to observation of critical times, and handwashing behavior at critical times, Bangladesh, 2007.

Our analysis consisted of three principal comparisons– 1) proportion of caregivers in the short versus long observation groups who had at least one event of each type observed (Figure 1), 2) overall number of events observed in the short observation group versus number of events expected, based on the events observed in the average 90 minutes of the long observation group (Figure 2) and, 3) handwashing events observed in the first 90 minutes in the long observation group versus handwashing events observed in the subsequent 210 minutes within the same group (Figure 2). Each of the following events were identified as an opportunity to wash hands: preparing food, serving food, feeding a child, eating, defecation, and cleaning a child who had defecated.

For the first comparison, we estimated risk difference (RD) to compare the proportion of caregivers who had at least one event of each type in the short observation group to that in the long observation group (Figure 1). Since start times varied by observation group and some types of events may be more frequent at particular times of day (e.g. defecation early in the day, cooking in mid-day), we introduced into the regression model a categorical variable denoting the start time of the observation. The start time variable was coded as 0 if the observation started between 8:00 am to 9:00 am, as 1 if the observation started between 9:01 to 10:00, and so on.

For the second comparison, we first calculated the mean number of observed events in long observation households. To derive the number of events expected in a 90-minute period, we divided the total number of events observed at each household in the long observation group by 3.33 (the ratio of 5 hours to 90 minutes). Then we compared the mean number of events expected and observed in 90-minutes period using linear regression, with the start time variable included in the model. We report p-values to describe the significance of differences between the number of expected events to the number of observed events in the short observation group in the linear regression model including the start time variable (Figure 2).

For the third comparison, within the long observation households, we assessed whether handwashing behavior observed in the first 90 minutes was associated with handwashing behavior observed during the subsequent 210 minutes (Figure 2). We used the number of handwashing events observed during the first 90 minutes to calculate the expected number of handwashing events during the subsequent 210 minutes. We then used paired t-tests to assess the significance of the difference between the number of events expected and observed during the subsequent 210 minutes. As part of the larger study, we assessed the reactivity of structured observations using bar soap containing an accelerometer [9]. Since these bar soaps were provided only to the long observation households, and respondents’ awareness of the accelerometers may have impacted handwashing behavior, we have not compared short and long observation households with respect to handwashing behavior.

## Results

Fifty caregivers each were enrolled into the long observation and short observation groups. Observation of one long observation household was not possible because of absence of the primary caregiver and her child during the scheduled day. The field team started long observations between 9:07 am and 11:09 am. The field team started observations between 9:00 am and 12:00 noon in 68% (34) of short observation households. Starting times ranged from 8:18 to 11:43 am for the first set of short observations and 11:45 am to 1:03 pm for the second set of short observations. For comparison of short versus long observation groups (Figure 1), at least one critical event was detected for 93% of caregivers in both groups (Table 1). Observation of at least one critical event was much higher in the long observation group than in the short observation group.

**Table 1:** Proportion of households with at least one observed event of critical times for handwashing, by duration of observations

**Variables**	**Long observation**	**Short observation**	P-value*
	**(n = 49)**	**(n = 50)**	
Proportion of households with at least one observed event of a critical time	100	96	0.061
Proportion of households with at least one observed food preparation event	78	34	0.001
Proportion of households with at least one observed food serving event	78	50	0.120
Proportion of households with at least one observed eating event	84	20	0.000
Proportion of households with at least one observed feeding event	92	58	0.001
Proportion of households with at least one observed defecation event	6	0	0.057
Proportion of households with at least one observed event of cleaning a child who had defecated	45	8	0.001

*Estimation of significance of difference between long and short observation households takes start time of observation into account.

We compared the mean number of events observed in the short observation group, to the expected number (as estimated using data from long observation households) for each of the events of interest (Table 2). The mean numbers of observed events of food preparation, food serving and feeding children were higher than the expected number, but differences between observed and expected numbers were not statistically significant(food preparation: p = 0.38; food serving: p = 0.12; child feeding: p = 0.61). The mean number of observed of eating (p < 0.05) and defecation (p = 0.06) events were lower than expected. There was no significant difference between the mean number of observed and number of expected events of cleaning children who had defecated (observed mean = 0.25, expected mean = 0.22; p = 0.52).

**Table 2:** Comparison of observed and expected number of critical events for handwashing during structured observation (SO) among households during a 90-minute observation, rural Bangladesh, 2007

**Variables**	**Observed in long observation households (unadjusted)**	**Expected in short observation households (adjusted)**	**Observed in short observation households (adjusted)**	**P-value**
	**(n = 49)**		**(n = 50)**	
Mean number of events(all types)	6.7 (SE 0.37)	1.91	2.0 (SE 0.62)	0.78
Mean number of food preparation events	1.0 (SE 0.10)	0.14	0.25 (SE 0.23)	0.38
Mean number of food serving events	1.3 (SE 0.15)	0.27	0.5 (SE 0.28)	0.12
Mean number of eating events	1.3 (SE 0.13)	0.45	0.25 (SE 0.18)	0.04
Mean number of child feeding events	2.3 (SE 0.20)	0.64	0.75 (SE 0.40)	0.61
Mean number of defecation events	0.06 (SE 0.03)	0.03	0.00 (SE 0.03)	0.06
Mean number of events of cleaning a child who defecated	0.61 (SE 0.12)	0.22	0.25 (SD 0.10)	0.52

Overall and at the following critical events, observed handwashing with soap was less frequent than expected in the latter 210 minutes of the structured observation: before food preparation, before eating, before feeding a child, and after cleaning a child who had defecated (Figure 2 and Table 3). No defecation event was recorded in the initial 90 minutes of long observation group and, thus, we could not compare observed handwashing after defecation to expected. Notably, observed handwashing with or without soap was more frequent than expected in the latter 210 minutes for all critical times, except for feeding a child.

**Table 3:** Comparison of observed handwashing during the latter 210 minutes of observation, compared to expected handwashing based on observed behavior during the first 90 minutes of 5-hour structured observations, rural Bangladesh, 2007

**Variables**	**Observed in the first 90 minutes (n = 49)**	**Expected in the remaining 210 minutes**	**Observed in the remaining 210 minutes (n = 49)**	**P-value**
Mean number of events (all critical times)	1.84 (SE 0.18)	4.29 (SE 0.43)	4.84 (SE 0.31)	0.292
Events when hands were washed*	1.31 (SE 0.18)	3.05 (SE 0.41	3.82 (SE 0.27)	0.126
Events when hands were washed with soap	0.24 (SE 0.07)	0.57 (SE 0.16)	0.55 (SE 0.11)	0.917
Mean number of before food preparation events:				
All events	0.22 (SE 0.07)	0.52 (SE 0.16)	0.80 (SE 0.09)	0.171
Events when hands were washed*	0.20 (SE 0.07)	0.48 (SE 0.15)	0.73 (SE 0.09)	0.179
Events when hands were washed with soap	0.02 (SE 0.02)	0.05 (SE 0.05)	0.02 (SE 0.02)	0.605
Mean number of before food serving events:				
All events	0.24 (SE 0.06)	0.57 (SE 0.14)	1.08 (SE 0.14)	0.017
Events when hands were washed*	0.22 (SE 0.06)	0.52 (SE 0.14)	1.00 (SE 0.13)	0.016
Events when hands were washed with soap	0.02 (SE 0.02)	0.05 (SE 0.05)	0.12 (SE 0.05)	0.283
Mean number of before eating events:				
All events	0.31 (SE 0.09)	0.71 (SE 0.21)	1.00 (SE 0.11)	0.254
Events when hands were washed*	0.26 (SE 0.07)	0.62 (SE 0.16)	0.75 (SE 0.08)	0.481
Events when hands were washed with soap	0.02 (SE 0.02)	0.05 (SE 0.05)	0.04 (SE 0.03)	0.904
Mean number of events before feeding a child:				
All events	0.90 (SE 0.11)	2.09 (SE 0.27)	1.45 (SE 0.17)	0.045
Events when hands were washed*	0.45 (SE 0.10)	1.05 (SE 0.23)	0.86 (SE 0.14)	0.483
Events when hands were washed with soap	0.14 (SE 0.06)	0.24 (SE 0.10)	0.14 (SE 0.06)	0.412
Mean number of defecation events:				
All events	0.0	0.0	0.06 (SE 0.03)	0.083
Events when hands were washed*	0.0	0.0	0.06 (SE 0.03)	0.083
Events when hands were washed with soap	0.0	0.0	0.06 (SE 0.034)	0.083
Mean number of events of cleaning a child who defecated:				
All events	0.16 (SE 0.05)	0.38 (SE 0.12)	0.45 (SE 0.10)	0.655
Events when hands were washed*	0.16 (SE 0.05)	0.38 (SE 0.12)	0.41 (SE 0.09)	0.860
Events when hands were washed with soap	0.08 (SE 0.04)	0.19 (SE 0.09)	0.16 (SE 0.12)	0.816

* Washed hands with water and with or without soap, ash or mud.

## Discussion

The findings of this study suggest that decreasing the duration of structured observation to 90 minutes from longer durations disproportionately reduces the opportunity to measure a number of critical events relevant to pathogen transmission, particularly fecal contact and eating. Since fecal contact and eating are frequently targeted critical times for handwashing with soap by public health programs, and since observed handwashing with soap after defecation during structured observation has been associated with reduced risk of child diarrhea [10], the inability to measure handwashing behavior at these critical times is an important drawback to short duration observations. During the latter 210 minutes of structured observation, we found handwashing with soap at several critical times was less frequent than expected based on behavior observed during the first 90 minutes, although statistical significance was not demonstrated likely due to the small sample size. However, during the latter part of the observation, we observed more frequent handwashing with water alone than expected before food preparation and food serving events. There is a substantial need for structured observations to capture accurately all types of hand cleansing behavior, e.g. handwashing with water alone or with soap and water, in order to accurately estimate the protective effects of hand hygiene for health outcomes.

Short duration observations may have yielded fewer fecal contact events than in long observation households for several reasons. First, observations in all households began after 8 am, a start time that was convenient because of the logistical barriers in transporting data collectors to field sites. In many communities, defecation by adults most commonly occurs early in the morning [11].Caregivers in the short observation households may not have needed to use the toilet during the short duration of the observation. As evidenced by the low number of defecation events even in the long observation households, it is possible that caregivers delayed toileting until the conclusion of the observation, out of embarrassment or because of an effort to be courteous to the observer, who may have been seen as a guest in the home. There was no difference between the expected and observed numbers of other types of critical times, such as food preparation and child feeding, which are likely not considered private behaviors in rural Bangladesh. Also, the start times of our structured observations may have led us to make both long and short observations during the mid-day meal preparation typical to rural Bangladesh. But, the lower number of eating events observed than expected in the short observation group may have been due to the break between the first and second observations occurring during lunch time.

Even with the limited statistical power of our study, soap use for handwashing was more common for each of the critical times except serving food in the first 90 minutes than in the subsequent 210 minutes among long observation households. Individuals react to the presence of outsiders in their home environment, as previously documented by our group and others with respect to structured observation of handwashing behavior [6,9]. Soap use may be especially prone to reactivity in the early part of an observation since the observer is newly present in the household environment. As household members become accustomed to the presence of the observer, they may revert to their typical handwashing behavior. Indeed, we find that they revert to handwashing with water, as evidenced by the observation of more frequent handwashing with water alone than expected during the latter 210 minutes suggests. If analysis of data from larger studies confirms our findings, there are two important implications for the use of structured observation for measurement of handwashing behavior. First, the behavior observed during short duration observations is likely compromised by reactivity, since household members may not become accustomed to the presence of the observer during the brief time that the observer is present. Second, data from the early part of a long duration observation may be sufficiently compromised by reactivity so as to warrant exclusion from analysis; researchers may wish to consider limiting analysis of structured observation data to soap use behavior from the latter part of the observation. In larger samples of structured observation data than available to us here, it would be worthwhile to investigate the patterns of reactivity over the course of structured observation in order to identify the period of observation when reactivity is minimal.

We propose that the optimal time for structured observations depends on objective of the study. In the rural Bangladeshi context, we recommend, the initiation of observation early in the morning, for example 5:00 am, if the principal study objective is to capture handwashing behavior after defecation events. Alternatively, if the intent is to observe handwashing at times of food preparation, mid-morning and mid-day might be the best times to be present in the home.

A limitation of this study was the lack of a uniform starting time within all households. Starting times varied because, in the short observation group, we intentionally instructed field workers to carry out two short duration observations per day in order to evaluate the efficiency of these shorter data collection times. Because of their location at disparate remote rural sites, starting times were typically in the mid-morning, which may have prevented the observation of defecation events. However, the mid-morning start times may have increased the likelihood of observing food preparation and food serving events. Because heavy monsoon rains and flooding in July and August 2007 disrupted and delayed field staff travel to the study areas, we were unable to maintain a uniform starting time across all study households. In our analyses comparing short and long observation households, we have attempted to adjust for the effect of the variation in start time. For an individual data collector, we ensured a minimum of one hour gap between two consecutive short observations. However, since some data collectors started the first observation of the day much earlier than others, there was some overlap in the time frames of first and second short observations. Since the duration of observation was the same for all the short observations, the overlap in time should not have substantially affected on the number of events observed.

Our study sample represents only a handful of communities in rural Bangladesh, and, thus, the generalizability of these findings is limited. However, as handwashing is increasingly promoted, analyses such as ours may assist investigators to design appropriate and feasible data collection approaches to evaluating the effects of promotion on handwashing behavior.

## Conclusions

Our findings suggest that compared with long duration observations, short structured observations lead to disproportionately fewer observations of critical events and an increased risk in measuring biased handwashing behavior. Future research should inform the development and evaluation of novel methods to reduce reactivity and improve the measures of handwashing behavior.

## Competing interests

The authors declare that they have no competing interests.

## Authors’ contributions

Conception and design of the study: AKH, PKR, SPL, JWM; Analysis and interpretation of data: AKH, PKR; Writing paper: AKH, PKR; Contribution to reagents/materials: not applicable. All authors read and approved the final manuscript.

## Pre-publication history

The pre-publication history for this paper can be accessed here:

http://www.biomedcentral.com/1471-2458/13/705/prepub
